# Finite Element Reconstruction of a Mandibular First Molar

**Published:** 2013-05-01

**Authors:** Sara Ehsani, Fatemeh Sadat Mirhashemi, Saeed Asgary

**Affiliations:** 1 Dental Research Center, Research Institute of Dental Sciences, Shahid Beheshti University of Medical Sciences, Tehran, Iran; 2 Iranian Center for Endodontic Research, Research Institute of Dental Sciences, Shahid Beheshti University of Medical Sciences, Tehran, Iran

**Keywords:** Finite Element Method, Mandibular First Molar, Three-dimensional Modeling

## Abstract

**Introduction:**

Mandibular first molar is the most important tooth with complicated morphology. In finite element (FE) studies, investigators usually prefer to model anterior teeth with a simple and single straight root; it makes the results deviate from the actual case. The most complicated and time-consuming step in FE studies is modeling of the desired tooth, thus this study was performed to establish a finite element method (FEM) of reconstructing a mandibular first molar with the greatest precision.

**Materials and Methods:**

An extracted mandibular first molar was digitized, and then radiographed from different aspects to achieve its outer and inner morphology. The solid model of tooth and root canals were constructed according to this data as well as the anatomy of mandibular first molar described in the literature.

**Result:**

A three-dimensional model of mandibular first molar was created, giving special consideration to shape and root canal system dimensions.

**Conclusion:**

This model may constitute a basis for investigating the effect of different clinical situations on mandibular first molars in vitro, especially on its root canal system. The method described here seems feasible and reasonably precise foundation for investigations.

## 1. Introduction

Health care research is substantially different from other non-medical scientific fields; it has its own unique problems, a central one being ethical issues in live subjects. Moreover, it may be very expensive as well as impractical in many circumstances. For example, direct measurement of stresses and strains in tooth root canal walls is impossible because it is inaccessible. When these limitations are considered, virtual simulation techniques becomes desirable research method (1).

A well known method for calculating stress and strain distribution within complex structures is the finite element method (FEM) (2). It is a theoretical approach first used in aerospace, civil engineering, and the automotive industries. The FEM has been proved to produce results similar to other experimental methods such as photoelastic and strain-gauge studies (3). However, it is more comprehensive than the photoelastic technique (4), and it does not have the limitation of strain gauges, because it is not limited by fixture placement. In fact, the FEM provides more detailed and more controllable mechanical responses (4-7). One unique aspect of FEM is that the experiment can be repeated extensively and therefore progressively improve the idea/experiment in a virtual environment. In this way, the actual trial would employ the best possible method on the living subjects (1).

The use of finite element (FE) analysis in dental research has been developed during the last decades (8-10). It is considered to be a fast, accurate, and reliable alternative compared to in vivo and in vitro investigation methods (11-14). The basic idea of FEM is that after dividing a complex structure into many smaller and simpler elements, strain and stress distributions can be assessed for every created element in response to different loading conditions. The deformation of the entire structure can be calculated by considering the individual deformations (1). But it seems that because of some practical complications, dental sciences take little advantage of this method (1). One of these limitations may be the intricacy of dental structures which makes their reconstruction time-consuming and costly.

A chief concern in clinical dentistry is the biomechanical breakdown of teeth after a number of dental treatments, which ends in vertical root fracture (VRF). VRF is a relatively hopeless clinical circumstance; extraction and currently, root amputation are the only possible treatments (15-17). This has encouraged some researchers to study mechanical response of teeth after various dental treatments that are thought to cause mechanical failures. Moreover, there has been a rapid development of radical techniques and restorations for extremely damaged teeth which would otherwise have been extracted. Consequently, awareness of stress distribution is of great clinical value (18).

Clinically, the mandibular first molar may be considered the most significant tooth. Because of its early eruption, it may require restoration more frequently than any other tooth. In addition, VRF is most frequently encountered in mandibular first molars (19). Despite its unique characteristics, most stress distribution investigations refuse to model this tooth due to its more complicated morphology and therefore modeling. Instead they prefer to select an anterior tooth with one single straight root. In addition, a straight canal is more favorable because the changes are more predictable (20).

It has been postulated that the most time-consuming and complicated step in a FE study is the modeling of the desired tooth (1). Due to the existent difficulties and complexities in this step, there is the temptation to simplify the model making the results and conclusions unrepresentative of the real case. The validity of finite element analysis is strongly dependent on model construction and its accuracy. However, little attention has been paid to the construction of a mimic model which expresses the morphological structure of dental anatomy. From this standpoint, development of a mimic model is critical for better interpretation FE results and study (21).

This FE study was carried out to meticulously reconstruct a mandibular first molar with the great precision for use in future studies.

## 2. Material and Methods

**Figure 1.**
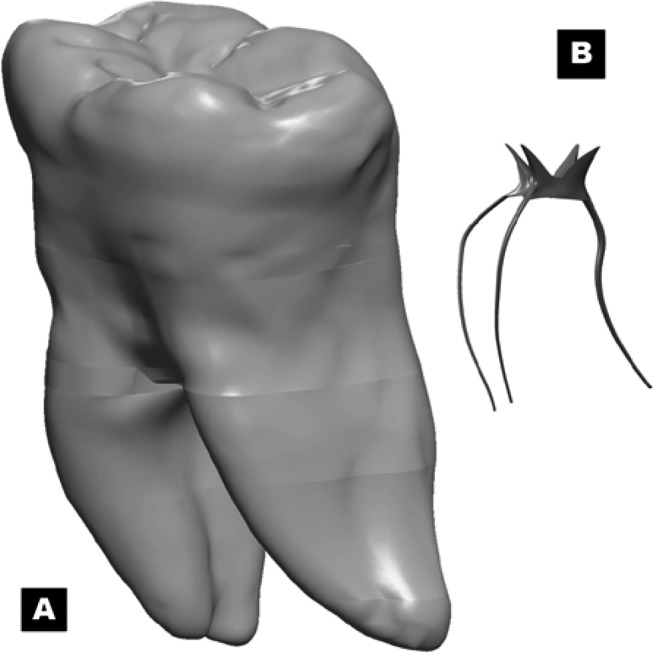
A) Solid model of mandibular first molar; B) 3D solid model of the lower 1^st^ molar root canal system

A mandibular first molar which was considered to be representative of the typical outer morphology was selected. The tooth was extracted for routine clinical purposes. Normally, a mandibular first molar has five well-developed cusps: two buccal, two lingual, and one distal cusp. It has also two buccolingually broad roots, one in mesial and one in distal side, which are widely separated at the apices (22). The external surface of the selected tooth was digitized using the optical digitizing system ATOS II (GOM, Braunschweig, Germany). The measured data was exported as a point cloud. From this cloud of points, a fine distribution of surface elements was generated using RapidForm software (INUS Technology, Seoul, Korea) to make a solid model of tooth. Then, the resemblance of the external geometry of the tooth model was checked with anatomy of the first molar described by Nelson & Ash (22) and necessary changes were made (Figure 1A). This process included two parts: the entire dimensions of the tooth were examined and then the tooth was divided into three sections (crown, midpart, and root). The final modification of each section was performed to achieve the most possible resemblance.

On the next stage, the solid model of the pulp system was generated using the literature data and the radiographs taken from the sample tooth. Normally, the pulp system of the mandibular first molar has three canals: one distal and two mesial canals. Two radiographs from two aspects (buccolingual and mesiodistal) were taken to make sure of the number and position of the canals. Afterward, the first molar was sectioned buccolingually through the centre of the tooth using a low-speed diamond disk (Leitz 1600 Microtome, Wetzler, Germany) to achieve visual information about position and curvature of the canals. The sections were radiographed on either side. These radiographs were scanned and imported into Mechanical AutoCAD software (AutoDesk Inc., San Raphael, California) to detect the boundaries of the pulp in each captured image. The contours, outlining the pulpal anatomy in 2 dimensions (2D), illustrated the path and curvatures of the pulp. These 2D contours were exported to SolidWorks software (SolidWorks 2006; SolidWorks Corp, Concord, Mass) to make a three-dimensional (3D) solid model of the pulp system (Figure 1B).

The geometry of root canal system has great variation in terms of diameter. Two basic canal shapes were constructed considering the typical canal shapes: two round canals (mesial) and one oval canal (distal). The canal diameter dimensions were assumed to be about 0.13 mm for the round canals and 0.20 mm for the oval canal at the apical foramen. The orifice dimensions were considered to be 0.40 mm mesiodistally and 0.80 mm buccolingually for the oval canal and 0.30 mm for the round canals, based on clinical experience. After the modifications, a model with a smooth surface and a mostly centered canal is completed (Figure 1B).

Subsequently, the 3D geometry of the enamel was made according to the anatomical guidance and the radiographs of the sample tooth. Then all these prepared models were assembled and finally an entire model of mandibular first molar was ready.

## 3. Results

**Figure 2.**
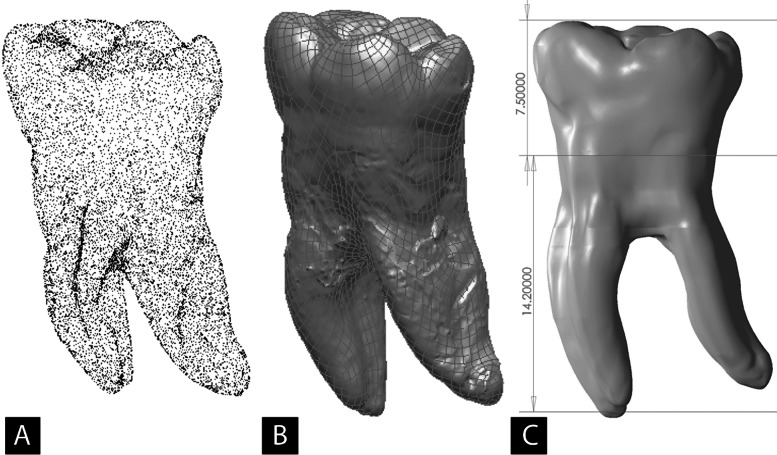
Construction procedure; A) The result of digitizing the extracted mandibular first molar; B) Fine distribution of surface elements generated using RapidForm software; C) The final model

A 3D model of mandibular first molar was created. Since the external surface was digitized by an optical digitizing system, we missed the least possible amount of data during this phase (Figure 2A). RapidForm software made it possible to adjust the number of surfaces passing from points cloud and resulted in a more precise model (Figure 2B). Finally, the model’s anatomy was checked to be an exact match of the first molar; hence, a detailed final model 3D finite element reproduction was constructed (Figure 2C).

## 4. Discussion

This paper describes a rather detailed method to create a finite element model of mandibular first molar as a base for future FE studies or other possible purposes, such as prototyping. This model may constitute a basis for future studies on mandibular first molar, and especially its root canal system.

No doubt, techniques which use micro CT are more precise and have greater detail. The model created in this study, however, represents a model that can be made in the absence of micro CT where it is unavailable. Maximum effort has been made to make this technique as precise as possible.

We achieved the 3D geometric model of the tooth by a 3D scanner (optical digitizing system). The precision of 3D scanners is considered to be more than that of Coordinate Measuring Machine (CMM) or conventional CT-Scan. The measuring error of CMM is more than 3D scanners because of contact measuring (touch-based measuring). The geometric model achieved by conventional CT-Scan images has more inaccuracies in comparison to 3D scanners because of the non clarity of the image edges (23). Therefore, we used a 3D scanner to have a more precise model with the most possible resemblance to the real tooth.

The choice of a 2- or 3-dimensional FE model is important (24). Two-dimensional FE analyses have long been performed (3, 18, 25), but three-dimensional dental models had been introduced in the 1990's (6, 8, 11, 26, 27), and have been successfully validated by experimental tests and clinical observations. Searching the literature reveals that the majority of FE studies are based on 2D models and only few cases had been performed on 3D ones. Although the construction of a 3D model is time consuming and costly, it yields more reliable results (24). Verification of the resemblance of external geometry of the tooth model with a typical anatomy and average dimensions made the model more generalizable.

Though small differences may perhaps exist between in vivo circumstances and the FE environment, this method is capable of displaying stress distribution within the complex root canal system which is impossible clinically (1). It should also be kept in mind that although the use of FEM provides very precise results, it estimates only a specific condition. Therefore, the FEM should not be expected to yield exact answers to real conditions but rather provides reliable estimates.

## 5. Conclusion

This model may constitute a foundation for future studies that investigate the effect of different situations on mandibular first molar, especially when studying the root canal system.
